# Supplementary material for: Single Seed Identification in Three Medicago Species via Multispectral Imaging Combined with Stacking Ensemble Learning
Source: Sensors (Basel). 2022 Oct 4;22(19):7521. doi: 10.3390/s22197521 (PMC9572871; doi:10.3390/s22197521)
Supplement: Supplementary file 1 [file sensors-22-07521-s001.zip › Supplementary tables.pdf]

Table S1. List of the extracted variables from multispectral images.

Name	Feature	Description
Area(mm^2)	Binary Feature	Area of blob.
Length(mm)	Binary Feature	Length of blob bounding box.
Width(mm)	Binary Feature	Width of blob bounding box.
Width/Length Ratio	Shape Feature	Ratio of width to length of the image oriented bounding box.
Compactness Circle	Shape Feature	Compactness of blob defined as 4*Area/(π *length^2), ratio of object area to the area of a circle with the same length (isolength quotient).
Compactness Ellipse	Shape feature	Compactness of blob defined as 4*Area/(π*length*width), ratio of object area to the area of an ellipse with the same length and width (isolength quotient).
BetaShape a	Shape Feature	Parameter a of beta-ellipse fitted to blob mask. Parameter a corresponds to width of most pointed blob-end.
BetaShape b	Shape Feature	Parameter b of beta-ellipse fitted to blob mask. Parameter b corresponds to width of least pointed blob-end.
Vertical orientation	Shape Feature	Skewness around the horizontal central axis.
Vertical skewness	Shape Feature	Vertical anisotropy index of characteristics.
CIELab L*	Color Feature	Mean Luminance component of CIELab-color of blob.
CIELab A*	Color Feature	Mean A-component of CIELab-color of blob.
CIELab B*	Color Feature	Mean B-component of CIELab-color of blob.
Saturation	Color Feature	Mean saturation of blob based on CIELab coordinates according to formulae: S = SQRT (A^2 + B^2).
Hue	Color Feature	Mean hue of blob based on CIELab coordinates according to formulae: H = ATAN (B/A).

Table S2. Mean spectral reflectance of alfalfa, hybrid alfalfa and sickle alfalfa.

	Spectral reflectance (%)		
Wavelength (nm)	*M. falcata*	*M. varia*	*M. sativa*
365	8.08a	7.1b	7.07b
405	8.59a	7.66b	7.61b
430	8.99a	8.01b	8.08b
450	10.58a	9.35c	9.88b
470	11.53a	10.32c	11.08b
490	12.18a	11.07b	11.88a
515	13.45a	12.46b	13.36a
540	13.87a	12.87b	13.76a
570	16.14b	15.05c	16.61a
590	17.72a	16.67b	18.2a
630	20.36a	19.07b	20.53a
645	21.35a	19.9b	21.34a
660	22.09a	20.27b	21.97a
690	25.03a	23.37b	25.01a
780	32.04b	31.53b	33.04a
850	41.21b	41.28b	43.01a
880	44.54b	44.74b	46.64a

940	51.83c	52.68b	55.00a
970	53.90c	55.00b	57.52a
