# Supplementary figures and images for: Single Seed Identification in Three Medicago Species via Multispectral Imaging Combined with Stacking Ensemble Learning
Source: Sensors (Basel). 2022 Oct 4;22(19):7521. doi: 10.3390/s22197521 (PMC9572871; doi:10.3390/s22197521)

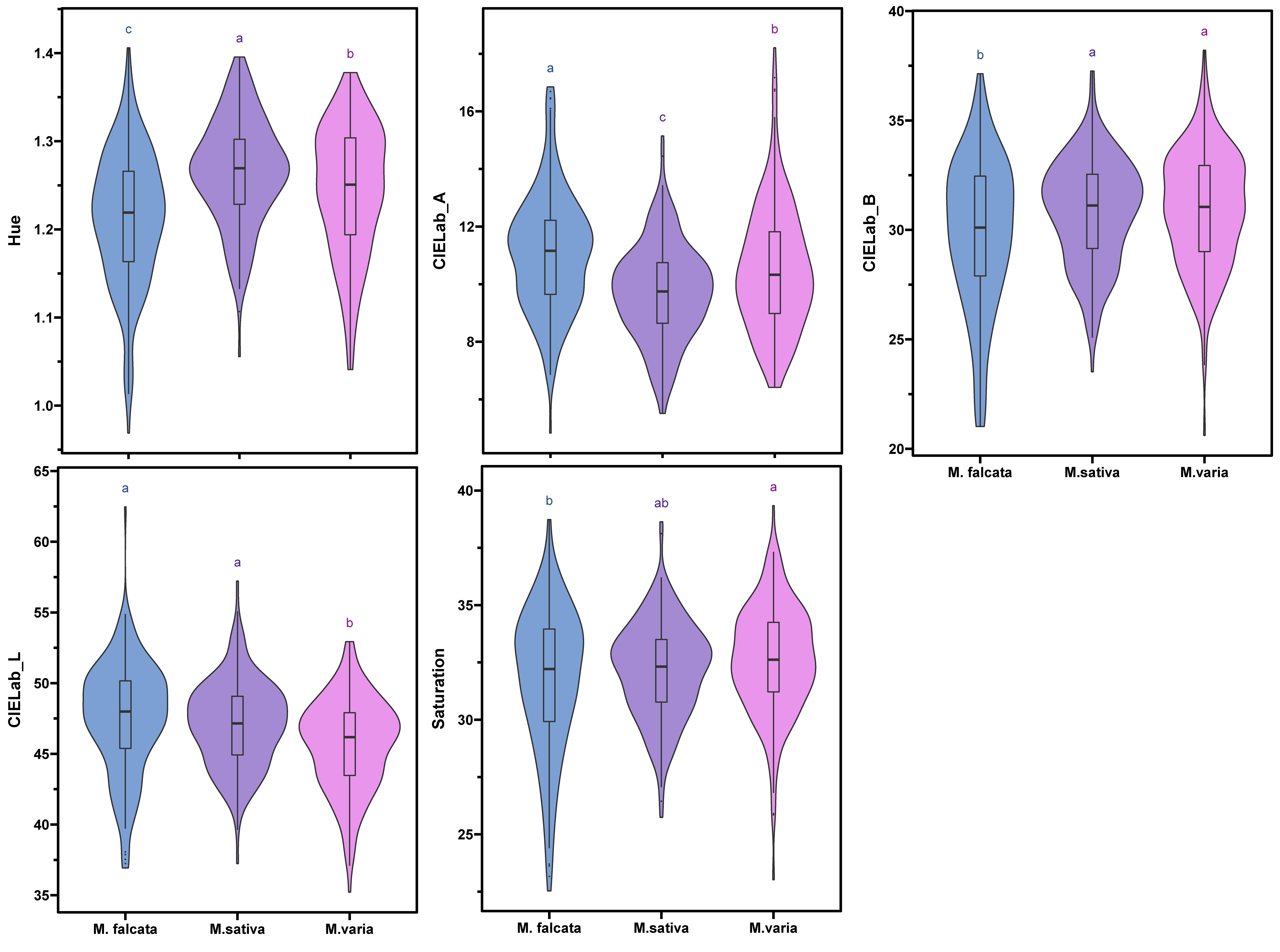

Supplement: Supplementary file 1 [file sensors-22-07521-s001.zip › Figure S3.tif]

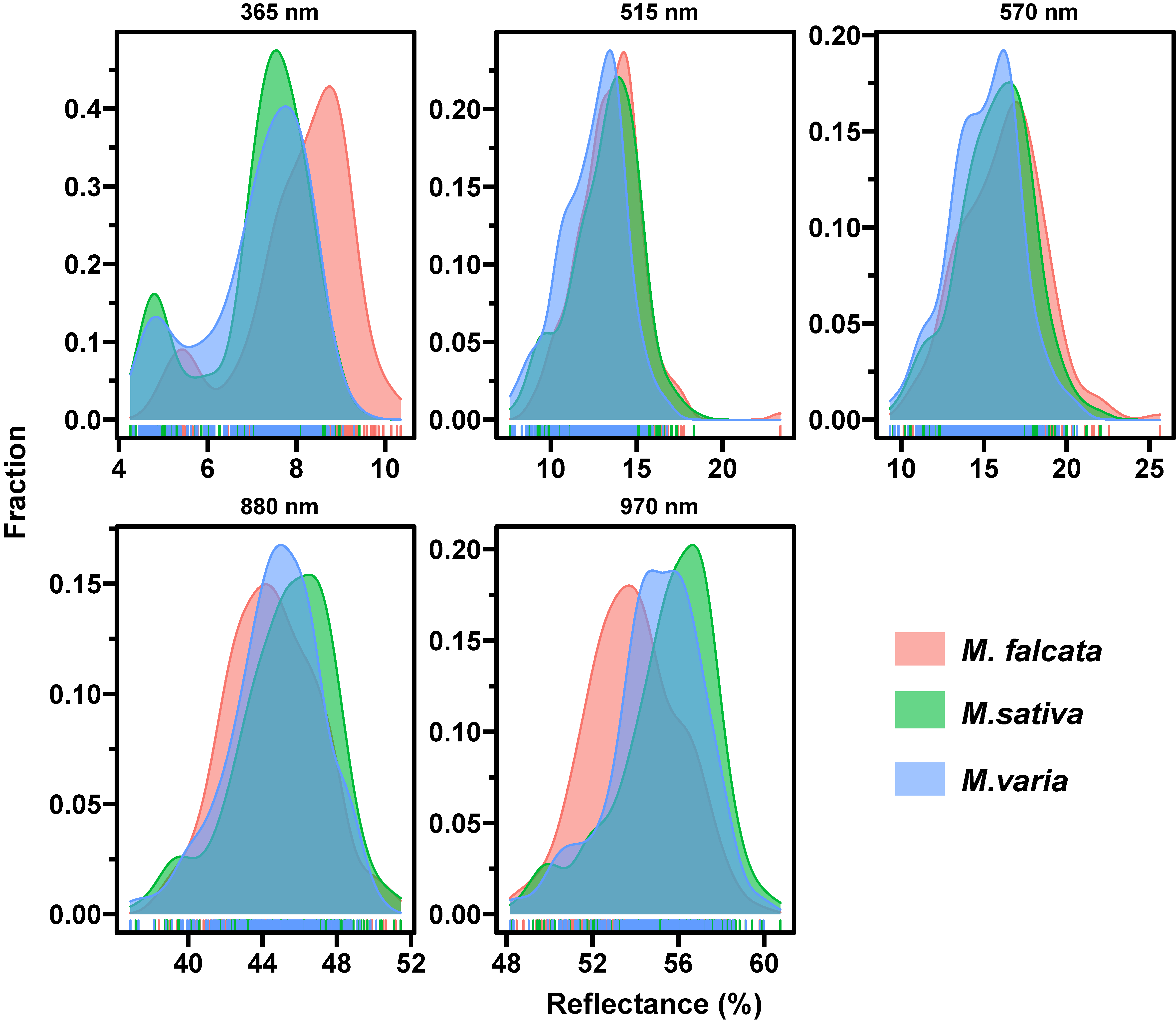

Supplement: Supplementary file 1 [file sensors-22-07521-s001.zip › Figure S4.tif]

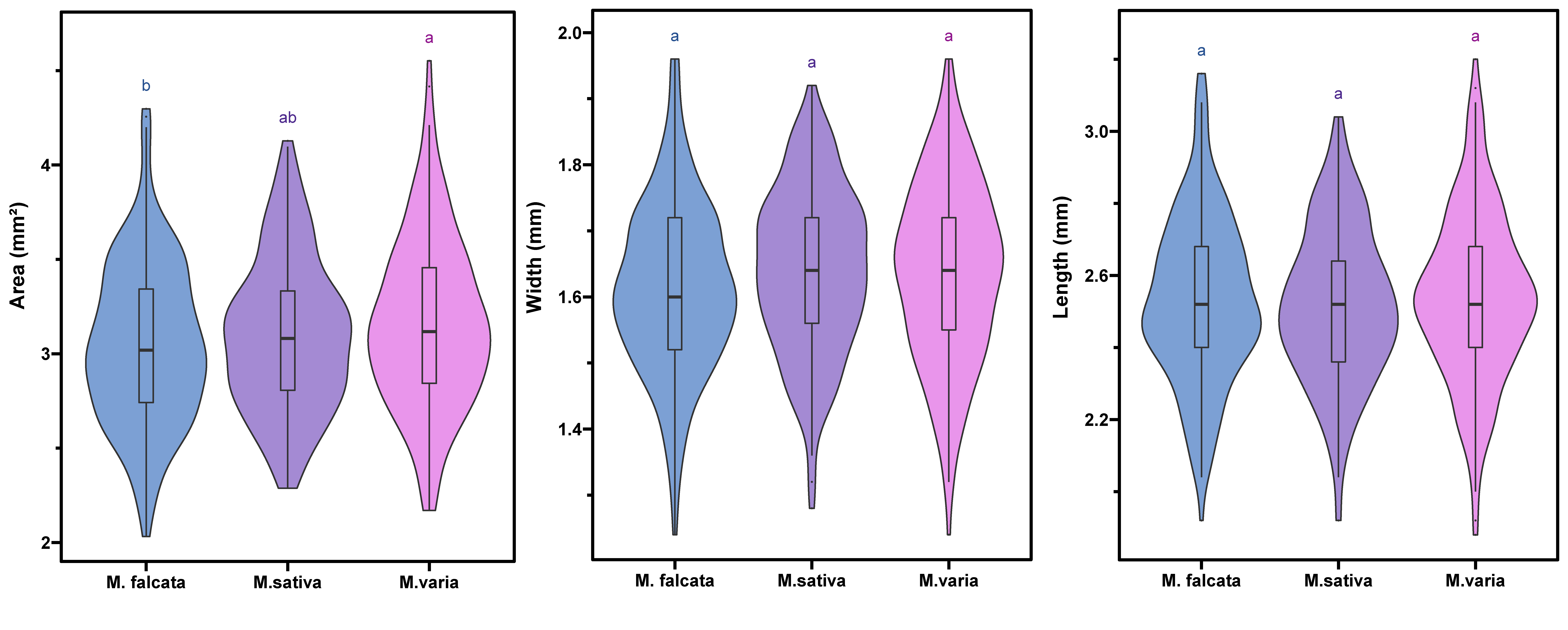

Supplement: Supplementary file 1 [file sensors-22-07521-s001.zip › Figure S1.tif]
